# Tissue-specific transcriptome analyses provide new insights into GPCR signalling in adult *Schistosoma mansoni*

**DOI:** 10.1371/journal.ppat.1006718

**Published:** 2018-01-18

**Authors:** Steffen Hahnel, Nic Wheeler, Zhigang Lu, Arporn Wangwiwatsin, Paul McVeigh, Aaron Maule, Matthew Berriman, Timothy Day, Paula Ribeiro, Christoph G. Grevelding

**Affiliations:** 1 Institute of Parasitology, BFS, Justus Liebig University, Giessen, Germany; 2 Department of Molecular Biosciences, Northwestern University, Evanston, Illinois, United States of America; 3 Department of Biomedical Sciences, Iowa State University, Ames, Iowa, United States of America; 4 Wellcome Trust Sanger Institute, Wellcome Genome Campus, Hinxton, United Kingdom; 5 The Institute for Global Food Security, School of Biological Sciences, Queen’s University, Belfast, United Kingdom; 6 Institute of Parasitology, McGill University, Montreal, Canada; University of Pennsylvania, UNITED STATES

## Abstract

Schistosomes are blood-dwelling trematodes with global impact on human and animal health. Because medical treatment is currently based on a single drug, praziquantel, there is urgent need for the development of alternative control strategies. The *Schistosoma mansoni* genome project provides a platform to study and connect the genetic repertoire of schistosomes to specific biological functions essential for successful parasitism. G protein–coupled receptors (GPCRs) form the largest superfamily of transmembrane receptors throughout the Eumetazoan phyla, including platyhelminths. Due to their involvement in diverse biological processes, their pharmacological importance, and proven druggability, GPCRs are promising targets for new anthelmintics. However, to identify candidate receptors, a more detailed understanding of the roles of GPCR signalling in schistosome biology is essential. An updated phylogenetic analysis of the *S*. *mansoni* GPCR genome (GPCR*ome*) is presented, facilitated by updated genome data that allowed a more precise annotation of GPCRs. Additionally, we review the current knowledge on GPCR signalling in this parasite and provide new insights into the potential roles of GPCRs in schistosome reproduction based on the findings of a recent tissue-specific transcriptomic study in paired and unpaired *S*. *mansoni*. According to the current analysis, GPCRs contribute to gonad-specific functions but also to nongonad, pairing-dependent processes. The latter may regulate gonad-unrelated functions during the multifaceted male–female interaction. Finally, we compare the schistosome GPCR*ome* to that of another parasitic trematode, *Fasciola*, and discuss the importance of GPCRs to basic and applied research. Phylogenetic analyses display GPCR diversity in free-living and parasitic platyhelminths and suggest diverse functions in schistosomes. Although their roles need to be substantiated by functional studies in the future, the data support the selection of GPCR candidates for basic and applied studies, invigorating the exploitation of this important receptor class for drug discovery against schistosomes but also other trematodes.

## Introduction

GPCRs form the largest known superfamily of transmembrane receptors in the Eumetazoa. They are involved in diverse biological processes, including growth, differentiation, neuronal signalling, olfaction, metabolism, and reproduction by interacting with different ligands such as neuropeptides, hormones, neurotransmitters, gases, volatile compounds, and biogenic amines. The importance of GPCRs is further reflected by their medical relevance because 30% to 50% of all pharmaceutical compounds target GPCRs and GPCR-mediated signalling pathways [1–4].

GPCRs possess seven alpha helices spanning the plasma membrane in a serpentine manner. While the N-terminus and extracellular loops are involved in ligand binding, the cytosolic parts and the C-terminus interact with downstream partners. Classically, GPCRs are defined as ligand-activated guanine nucleotide exchange factors (GEFs) for heterotrimeric guanine nucleotide-binding (G) proteins that transmit signals intracellularly by interacting with different effector molecules. This mostly results in the modulation of second messenger concentrations, which in turn provoke cellular responses [5]. In addition to this traditional view, research activities have led to a tremendous increase in knowledge on the versatility of GPCR signalling. Among other functions, GPCRs activate G protein–independent signalling pathways through adaptor proteins like arrestins [6,7], and they form homo- and hetero-oligomers or receptor mosaics consisting of three or more protomers [8,9]. Furthermore, GPCRs can cooperate with different membrane proteins such as integrins and receptor tyrosine kinases (RTKs) [10–13]. This functional flexibility, combined with the use of different downstream effector molecules such as signal transmitters, has fundamental consequences; it impacts receptor function and physiology, offering a platform for the diversification of signalling processes, regulation, crosstalk, internalization, trafficking, and GPCR pharmacology [9].

Several classification systems have been developed to divide the GPCR superfamily into subclasses. According to sequence similarities, the A–F system (used here) splits GPCRs into six main classes, in which class A (rhodopsin-like receptors), class B (secretin/adhesion receptors), class C (glutamate receptors), and class F (frizzled receptors) are the main classes in the Eumetazoa [5,14–17]. Among these, class A comprises the majority of all known GPCRs [16,18]. Additionally, some lineage-specific GPCR classes have been identified, such as the nematode chemosensory receptors [19] or insect gustatory receptors (which is still under debate [20]).

While much information on GPCR signalling exists for model organisms and higher vertebrates, our knowledge of platyhelminth GPCRs is still fragmentary. This invertebrate phylum includes free-living and parasitic flatworms like blood flukes of the genus *Schistosoma*. Schistosomes cause schistosomiasis, an infectious disease with tremendous impact on human health and socioeconomic development worldwide. Schistosomiasis is endemic in 76 countries, mainly in the developing regions of Africa, Asia, and America with over 230 million infected people [21]. A vaccine is not available, and controlling schistosomiasis relies on a single drug, praziquantel. Due to the prospect of emerging resistance, there is an urgent need to find alternative treatment strategies [22–24]. Understanding the biology of this parasite is central to the identification of candidate genes and/or proteins, which may serve as new targets for drug or vaccine development.

Because egg production is essential for life cycle completion and for triggering the pathological consequences of schistosomiasis [25], the unique reproduction biology of schistosomes is of particular interest [26,27]. As an exception among trematodes, schistosomes have evolved separate sexes. Adult male and female worms live constantly paired, a prerequisite for the development of the female gonads [26,27]. Pairing-inexperienced females (sF) are sexually immature and possess stem cell–like precursor vitelline cells and a small ovary containing stem cell–like precursor oocytes, the oogonia. Upon pairing, differentiation processes are induced, leading to the maturation of the ovary and vitellarium that characterizes a sexually mature female (bF). In contrast with females, pairing-inexperienced males (sM) possess testes with differentiated spermatocytes and exhibit no morphological differences from pairing-experienced males (bM) [28–31]. Nevertheless, pairing also induces changes in male gene expression [32–34].

Sequencing of the *S*. *mansoni* genome provided the basis for a variety of in silico analyses [35,36]. Among others, bioinformatics unravelled GPCRs as the largest superfamily of transmembrane receptors, and all major subfamilies were represented, including a platyhelminth-specific rhodopsin subfamily [37,38]. Although these findings emphasize the importance of GPCR signalling in schistosomes, only a few GPCRs have been functionally characterized. Most of these respond to classical biogenic amines and neurotransmitters like dopamine, serotonin, histamine, and acetylcholine. Using RNA interference (RNAi) or pharmacological antagonism, GPCR functions were associated with muscular activity in larval or adult worms [39–42]. Only a few studies linked schistosome GPCRs to other functions such as gametogenesis and embryogenesis [43]. Nevertheless, the diversity of GPCR genes in *S*. *mansoni* suggests a broad spectrum of different functions, potentially including reproduction. This hypothesis is supported by studies of the planarian *Schmidtea mediterranea* in which neuropeptide GPCRs with key roles in reproductive development were identified [44].

## An updated phylogenetic analysis of the *S*. *mansoni* GPCR*ome*

An updated analysis of the *S*. *mansoni* GPCR complement confirmed many patterns originally deduced from the initial description of the genome [37]. There remain 115 putative GPCRs with three or more predicted transmembrane domains (TMs), two less than originally suggested. Importantly, each receptor included here is linked to a gene model validated by previous whole transcriptome RNA sequencing (RNA-seq) experiments [36], indicating remarkable congruence with the original analysis that at the time had very few expressed sequence tags (ESTs) available. Using the new gene models, we were able to more precisely annotate some of these genes (S1 Table). Specifically, we reduced the subset of class A GPCRs, added one receptor to both class B and class C, and maintained the original count of class F receptors. Two receptors (Smp_049330, Smp_170350) escaped classification into any of the GPCR classes [17], both of which contain a Lung_7-TM domain (pfam06814) and one of which shows similarity to GPR107, an intracellular signalling receptor that localizes to the trans-Golgi network [45].

**Fig 1:**
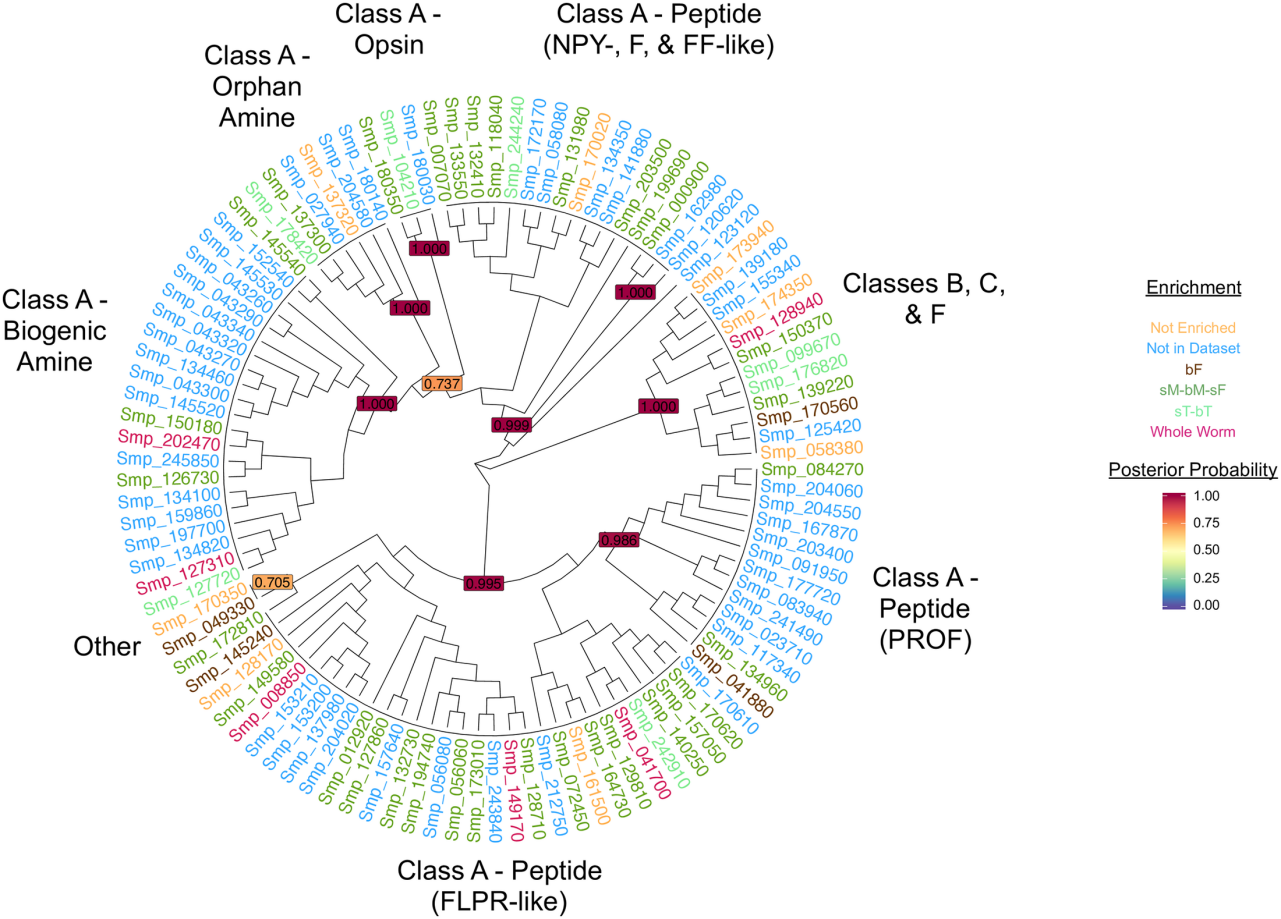
Phylogenetic analysis of *S*. *mansoni* GPCR genes. A Bayesian tree of putative *S*. *mansoni* GPCRs was inferred with the software tool MrBayes3.2 [92]. The Tree is rooted between class A and classes B, C, F, and others. Broad subclassifications are indicated, each corresponding to a highly supported node. Gene IDs are coloured according to transcriptomic enrichment. bF, pairing-experienced (bisex) females; bM, pairing-experienced (bisex) males; bT, testes from bM; FLPR, FMRFamide-like Peptide GPCR; GPCR, G protein–coupled receptor; PROF, Platyhelminth-Specific Rhodopsin-like Orphan-Family; sF, pairing-inexperienced (single-sex) females; sM, pairing-inexperienced (single-sex) males; sT, testes from sM.

We analysed the phylogeny of 105 of these putative GPCRs, only including those that had more than four predicted TMs in order to infer the highest confidence topology (Fig 1). The tree is rooted between class A and classes B, C, and F. The topology mimics phylogenies inferred from other organisms, showing that the class A aminergic receptors, which include orphan amines, biogenic amines, and opsins, evolved from a common, peptide receptor-like ancestor [46]. The putative peptidergic receptors split into three highly supported clades—one containing receptors similar to Neuropeptide Y (NPY), Neuropeptide F (NPF), and Neuropeptide FF (NPFF) GPCRs, one containing receptors similar to FMRFamide-like Peptide GPCRs (FLPRs), and a flatworm-specific clade containing GPCRs originally designated the Platyhelminth-Specific Rhodopsin-like Orphan-Family (PROF). The Lung_7-TM domain receptors were found to be most nearly related to the FLPRs. The PROF family has so far defied annotation, though some have suggested it shows similarity to an ancient family of chemoreceptors, the nematode Srw family [19,44]. However, unlike the *Caenorhabditis elegans* Srw family, of which 90% are concentrated on the same chromosome [47], the PROF orthologs of *S*. *mansoni* are spread throughout the genome (S1 Table).

## Transcriptomic data reveal new insights into GPCR function

**Fig 2:**
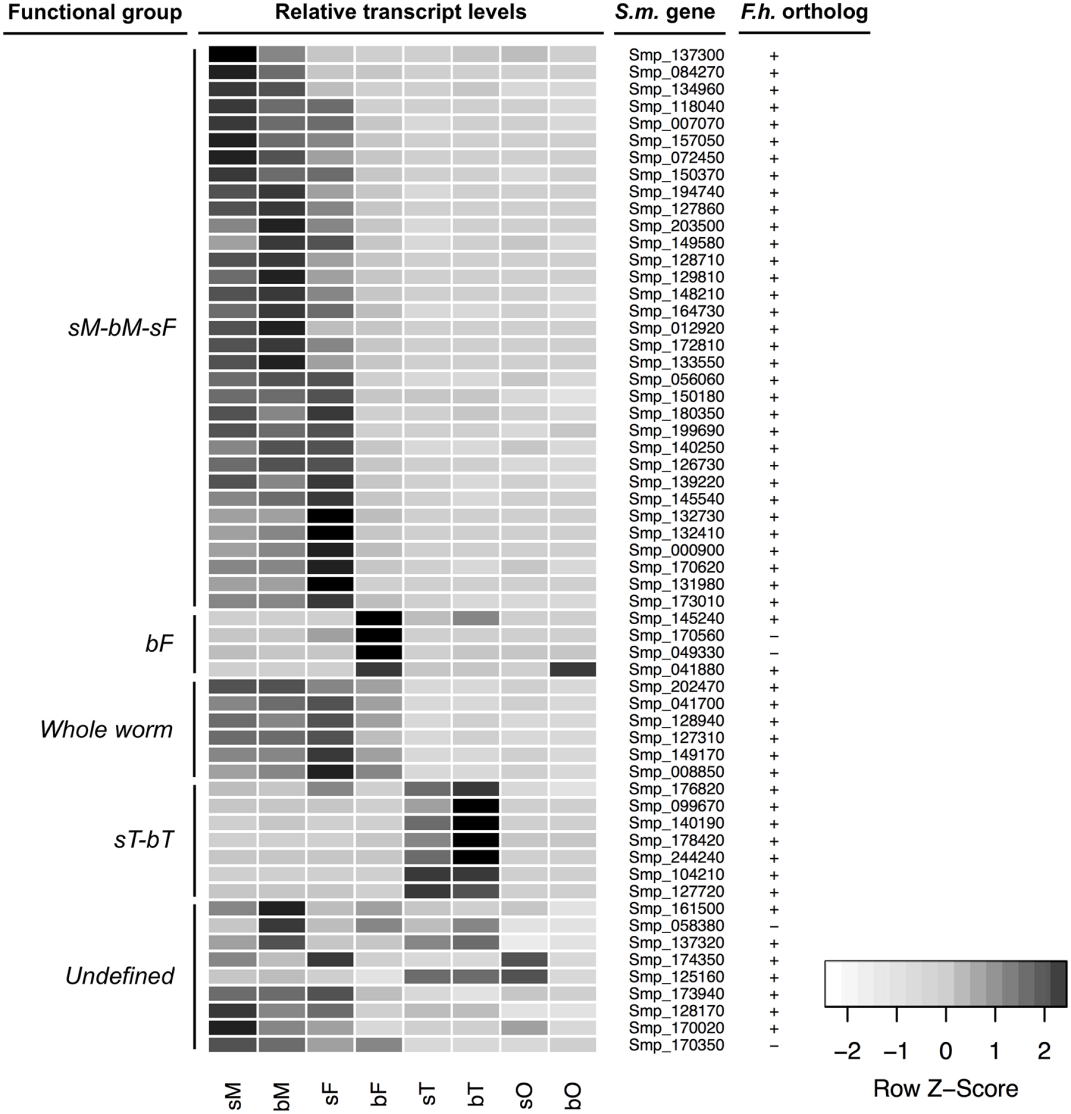
Hierarchical clustering of GPCR genes expressed in adult *S*. *mansoni*. Shown are functional groups to which GPCRs were assigned according to their relative transcript levels in adult *S*. *mansoni* obtained by a recent RNA-seq study from Lu et al. [32]. The heat map shows relative gene expression in all samples, which was calculated by the Z-score method implemented in the heatmap.2 function of the gplots package. Darker color indicates higher expression across all samples, and lighter color indicates lower expression levels (Color Key). The sM-bM-sF group contains GPCR genes with a transcription bias towards males and pairing-inexperienced females, while the “whole worm” group displays a balanced transcription rate among both sexes independently of pairing. Instead of this, the bF and sT-bT groups contain GPCRs that are preferentially transcribed in reproductive organs, namely testis, ovary, or vitellarium. *S*.*m*. gene provides Smp numbers for the listed GPCRs of *S*. *mansoni*. Existing/missing orthologs in *F*. *hepatica* (*F*. *h*.) are indicated by +/- (see S1 Table for details). sM, pairing-inexperienced (single-sex) males; bM, pairing-experienced (bisex) males; sF, pairing-inexperienced (single-sex) females; bF, pairing-experienced (bisex) females; sT, testes from sM; bT, testes from bM; sO, ovaries from sF; bO, ovaries from bF.

Based on progress in organ isolation from schistosomes [43,48], a comparative RNA-seq analysis on paired versus unpaired *S*. *mansoni* and their gonads recently unravelled sex-, tissue-, and pairing-dependent transcription patterns [32]. These data revealed that approximately 60% of the GPCR genes were expressed in adult *S*. *mansoni*, covering all classes of the phylogenetic analyses. With respect to the complex life cycle of schistosomes, which includes different larval stages, it was expected that part of the GPCR*ome* would not or only weakly be expressed in adults. Indeed, several missing GPCRs were linked to functions in the larval stages like the miracidium [49]. Additionally, transcriptome data obtained by a former RNA-seq study [36] indicate that most of the missing 47 GPCRs are less abundantly transcribed in adult worms compared with other life stages (S1 Fig). In addition, few GPCRs already functionally characterized in adults were also absent from the transcriptome data of Lu et al. [32] due to transcript levels below threshold. These included the amine receptors SmGPR-1 (Smp_043260), SmGPR-2 (Smp_043340), and SmGPR-3 (Smp_043290) that were shown to be expressed in the nervous system of adult worms [42,50,51]. In general, GPCRs are low-abundantly expressed in adult *S*. *mansoni* compared with other gene families, which is in accordance with findings from other organisms [52]. Focusing on GPCRs in adult *S*. *mansoni*, most exhibited sex-/pairing- and/or tissue-preferential transcription. These findings add to existing knowledge allowing the categorization of GPCRs into specific functional groups (Fig 2).

### Expression of GPCRs in nongonad tissues

Most GPCRs detected in the RNA-seq analysis were found to be mainly transcribed in nongonad tissues of both sexes. This included deorphanized neurotransmitter-activated GPCRs like the serotonin receptor Sm5THR (Smp_126730) that is expressed throughout the nervous system of adults and was shown to be involved in the control of muscle activity using RNAi [40]. The dopamine receptor SmD2 (Smp_127310) is expressed in the in subtegumental muscles and the muscular lining of the intestine [53]. SmGluR (Smp_128940) is phylogenetically related to metabotropic glutamate receptors and responsive to glutamate in ligand-binding assays. Immunolocalization revealed its expression throughout the nervous system of adult worms and along the female reproductive duct, including the oviduct, ootype, and uterus, whereas no expression was detected in the ovary and vitellarium [54]. Remarkably, for the majority of GPCRs expressed in nongonad tissue, a pairing-influenced transcript occurrence was observed, supporting recent data on the importance of neuronal processes in schistosome male–female interaction [32,33,55]. Moreover, because the transcript levels of most of these GPCRs showed a bias towards sM, bM, and sF (sM-bM-sF group; Fig 2), the data are in agreement with previous findings that the transcriptome of the sF seems to be more closely related to the male than to the bF [32]. These similarities in GPCR expression suggest that biological processes like locomotion or partner attraction have similar importance in sM, bM, and sF. Within this group, approximately 30% of the GPCRs dominate in one of the three subgroups (Fig 2), respectively. The GPCRs with the highest transcript levels in sF may contribute to processes in male attraction and/or repressing female maturation until pairing. In turn, highest transcript levels in sM suggest functions in locomotion and female attraction and/or perception. Those with the highest transcript levels in bM may indicate a higher need for neuronal processes associated with pairing and clasping processes and increased muscle activity for the successive female transport. Furthermore, the occurrence of GPCRs with sex-specific expression corresponds to findings from *C*. *elegans* and *Drosophila melanogaster*, for which it was shown that the nervous system regulates reproductive processes such as mating behavior, insemination, and fecundity in a sex-specific manner [56–62].

### Expression of GPCRs in the reproductive organs

Surprisingly, only a minority of GPCRs detected in the RNA-seq analysis exhibited a gonad-preferential expression in *S*. *mansoni*. Upon pairing, the schistosome female streamlines its biology towards reproduction, which is also reflected by a remarkable shift in gene expression [32,34]. However, only four GPCRs, all so far functionally uncharacterized in *S*. *mansoni*, showed a significant transcription bias towards bF (Smp_049330, Smp_145240, Smp_170560, Smp_041880; bF group; Fig 2). This indicates an abundant expression in the vitellarium, which is also supported by an independent study exploring subtranscriptomes of the bF [63]. The vitellarium is the most prevalent tissue in bF, producing S4 vitellocytes for the synthesis of composite eggs [27]. One candidate of this group (Smp_041880) represents the only GPCR with a significant tissue-preferential transcript profile in the ovary. Smp_041880 codes for an ortholog of a potential allatostatin receptor recently highlighted as important for reproductive development in *Schistosoma japonicum* females [55]; allatostatin family neuropeptides are insect and crustacean hormones involved in the generation of juvenile hormone, feeding, and reproduction [64]. Nevertheless, due to our phylogenetic analysis, Smp_041880 is grouped into the PROF subfamily of class A GPCRs, suggesting that flatworm-specific molecules might serve as natural ligands of this receptor. The other members of the bF group were phylogenetically classified as FLPR (Smp_145240), class B (secretin/adhesion) GPCR (Smp_170560), a class known to be regulated by peptide hormones [65], or they escaped classification (Smp_049330).

Seven GPCRs exhibited a testis-biased transcription (sT-bT group; Fig 2), including two class-B (secretin/adhesion) GPCRs (Smp_176820, Smp_099670) and an opsin receptor (Smp_104210). The latter displays testis-specific expression, which appears to contrast with the established light and circadian rhythm–associated functions of opsins [66,67]. However, within the vertebrate opsin family are neuropsins, which have been localized in eye, brain, spinal cord, and testes, although their functions are unknown [68]. The remaining GPCRs of this group can be connected to amine (Smp_127720, Smp_178420) and neuropeptide signalling (Smp_242910, Smp_244240), most notable as neuropeptides play a conserved role in reproduction in different model organisms, including *S*. *mediterranea* [44,69] and *D*. *melanogaster* [70–72]. In *S*. *mansoni*, the transcription profile of neuropeptides points to their participation in sex-specific processes in adults [32]. Remarkably, four GPCRs of this group (sT-bT) show higher transcript levels upon pairing in the testis, supporting previous data showing that molecular changes occur in the gonads of males after pairing [32,33,43]. This may reflect an increased sperm production upon mating.

An outstanding role might be fulfilled by a frizzled ortholog (Smp_174350) that appears to be highly expressed in the ovary of sF. Previous studies revealed that Wnt proteins bind to receptors of the Frizzled family, and they are involved in the control of various types of stem cells, acting as niche factors maintaining stem cells in a self-renewing state [73]. Because immature oocytes, including stem cell–like oogonia, predominate in the ovary of sF [27,28], it is tempting to hypothesize that Smp_174350 may play a role in a Wnt signalling pathway important for the maintenance of the immature state of the oocytes. In agreement with this hypothesis, one of the dishevelled orthologs in *S*. *mansoni* (Smp_020300.1) revealed a similar transcript profile [74]; dishevelled proteins are involved in canonical and noncanonical Wnt pathways [75,76].

## A glance at liver fluke GPCRs

Temperate (*F*. *hepatica*) and tropical (*F*. *gigantica*) liver fluke species have a profound impact on livestock animals globally [77,78] as well as represent a zoonotic threat to human health [79]. The recent publication of the *F*. *hepatica* genome [80] has facilitated comparative analyses of its GPCR complement, although tissue-specific expression data are not yet available for this species or other liver flukes. Comparisons of GPCR complements between the hermaphroditic liver fluke and dioecious schistosomes could allow interpretation of conserved and species-specific GPCR functions given the distinct biology of these trematode lineages. Using hidden Markov models (HMMs) trained against the originally published *S*. *mansoni* and *S*. *mediterranea* GPCR complements, 147 GPCRs were identified in the *F*. *hepatica* genome. These comprise 136 rhodopsin-like (class A), two adhesion (class B), and three metabotropic glutamate receptors (class C), with five frizzled and a single smoothened GPCR representing class F. Phylogenetically, the class A receptors comprise 40 aminergic receptors, two photo-activated opsins, and 94 putative peptide receptors. While many class A GPCRs have readily identifiable orthologs in model systems permitting the assignment of putative ligands, they also include groups lacking obvious nonflatworm orthologs that appear to be expanded within flatworm lineages. The latter includes six putative PROF1 receptors showing highest identities with existing PROF1s from S. *mansoni*, *S*. *mediterranea*, and *Echinococcus multilocularis*. At least two other clades appear to be expanded within the Echinostomatoidea lineage; phylogenetic reconstructions of these receptor clades suggest the absence of obvious nonflatworm orthologs (personal communication from P. McVeigh and A. Maule). A first comparison of both GPCR*omes* revealed that most schistosome GPCRs share orthologs with *F*. *hepatica*, while six GPCRs turned out to be *S*. *mansoni*-specific. Overall, 55 of the 59 GPCRs shown to be transcribed in adult schistosomes display homology to *F*. *hepatica* genes. According to transcriptome data available for different life stages of this parasite [80], most of those orthologs are transcribed in adult liver flukes as well.

Because there is a lack of tissue-specific analyses for *F*. *hepatica*, it seemed reasonable to take a closer look at GPCRs with a gonad-preferential transcription in *S*. *mansoni*. In total, nine of the eleven *S*. *mansoni* GPCRs categorized to the bF and sT-bT groups have orthologs in *F*. *hepatica*, but for only seven of them was transcription proven for the adult stage. While the detection of two testis-preferentially–transcribed GPCRs might have failed due to the absence of gonad-specific transcriptome data in liver flukes, both orthologs of the bF group are among the most abundantly transcribed GPCRs in adult *F*. *hepatica* and female *S*. *mansoni*. Because of the relevance of the vitellarium and the ovary in the egg-laying stages, this might indicate a common function of these GPCRs in both parasites. In combination with the transcription of at least five orthologs of the *S*. *mansoni* sT-bT group in adult liver flukes, these findings provide first evidence for conserved GPCR signalling pathways controlling gametogenesis and vitellogenesis in different parasitic flatworm species (Fig 2; S1 Table; S2 Fig).

## GPCRs as potential drug targets

Besides transmitter-activated ion channels [81,82], GPCRs are a focus for research activities centered on helminth neurochemistry and the identification of druggable targets for the development of new anthelmintics [37,41,83–85]. Their role as privileged candidates originates from functional studies and drug-modelling approaches that have been well established for this receptor class. GPCRs reveal a large diversity among species and can bind distinct ligands, which allows the development of tailor-made compounds that reduce the possibility of host toxicity [86–91]. Among the candidate GPCRs in *S*. *mansoni* is the serotonergic GPCR (Sm5HTR; Smp_126730) [91], a member of the sM-bM-sF group, which is predominantly transcribed in males independent of pairing [32,74]. In bF, its transcription was found to be reduced by about 60% compared with sF. Through pharmacological profiling, new ligands and chemical series have been found, which are selective for Sm5HTR over Hs5HTR7, the closest human GPCR ortholog. The identified compounds, such as nuciferine, showed efficacy against adult worms and schistosomula in vitro as well as in vivo, and evidence was obtained that Sm5HTR was irreversibly inactivated [90,91]. These results demonstrate the potential for schistosome GPCRs as targets for new anthelmintics.

## Conclusions

Schistosomes as well as other trematodes have a tremendous impact on global health and socioeconomic development. In the face of emerging resistance against commonly used therapeutics, alternative drug targets are needed to support the development of next-generation anthelmintics. To this end, basic research has the challenging task of connecting the improving genome data with biological functions to identify key pathways and molecules essential to parasite biology. With respect to their fundamental role throughout the Eumetazoa and their pharmacological importance, GPCRs represent promising candidate targets for parasite control. Phylogenetic analyses display their diversity in free-living and parasitic platyhelminths and suggest diverse functions in schistosomes. Whilst these receptors have been shown to play a role in neuronal processes and locomotion in adults and larval stages of *S*. *mansoni*, tissue-specific transcriptome analyses have provided new insights into their participation in its unique reproduction biology. These new resources not only support the identification of GPCRs with gonad-specific expression profiles but also reveal that the majority of GPCRs have a nongonad but pairing-dependent expression profile. While GPCRs of the first group might play roles in gametogenesis, vitellogenesis, and embryogenesis, the latter may contribute to the complex male–female interaction. Beyond this, orthologs of some of these GPCRs can be found in the genome of the liver fluke *F*. *hepatica*, opening the possibility of studying conserved GPCR signalling pathways in trematode reproduction as well as how GPCRs may differentially function in trematodes with divergent sexual characteristics. Although these preliminary findings need to be substantiated by functional studies in the future, the data support the selection of candidate receptors for basic and applied studies, invigorating the exploitation of this important receptor class for the discovery of drugs to control schistosomes and other trematodes.

## Supporting information

S1 Table Updated list of GPCR genes in *S*. *mansoni*, their average expression values according to RNA-seq, and predicted orthologs in *F*. *hepatica*. GPCR, G protein–coupled receptor.
(XLSX)

S1 Fig Relative expression of GPCRs absent of the Lu et al. dataset [32] among different *S*. *mansoni* and *F*. *hepatica* life stages. GPCR, G protein–coupled receptor.
(PDF)

S2 Fig Relative expression of GPCR orthologs in adult stages of *F*. *hepatica* and *S*. *mansoni*. GPCR, G protein–coupled receptor.
(PDF)
