# Supplementary material for: Tissue-specific transcriptome analyses provide new insights into GPCR signalling in adult Schistosoma mansoni
Source: PLoS Pathog. 2018 Jan 18;14(1):e1006718. doi: 10.1371/journal.ppat.1006718 (PMC5773224; doi:10.1371/journal.ppat.1006718)
Supplement: S1 Fig — GPCR, G protein–coupled receptor. (PDF) [file ppat.1006718.s002.pdf]

Figure S1: GPCRs not found in the underlying RNA-seq dataset are lowly expressed in *S. mansoni* adults.

A

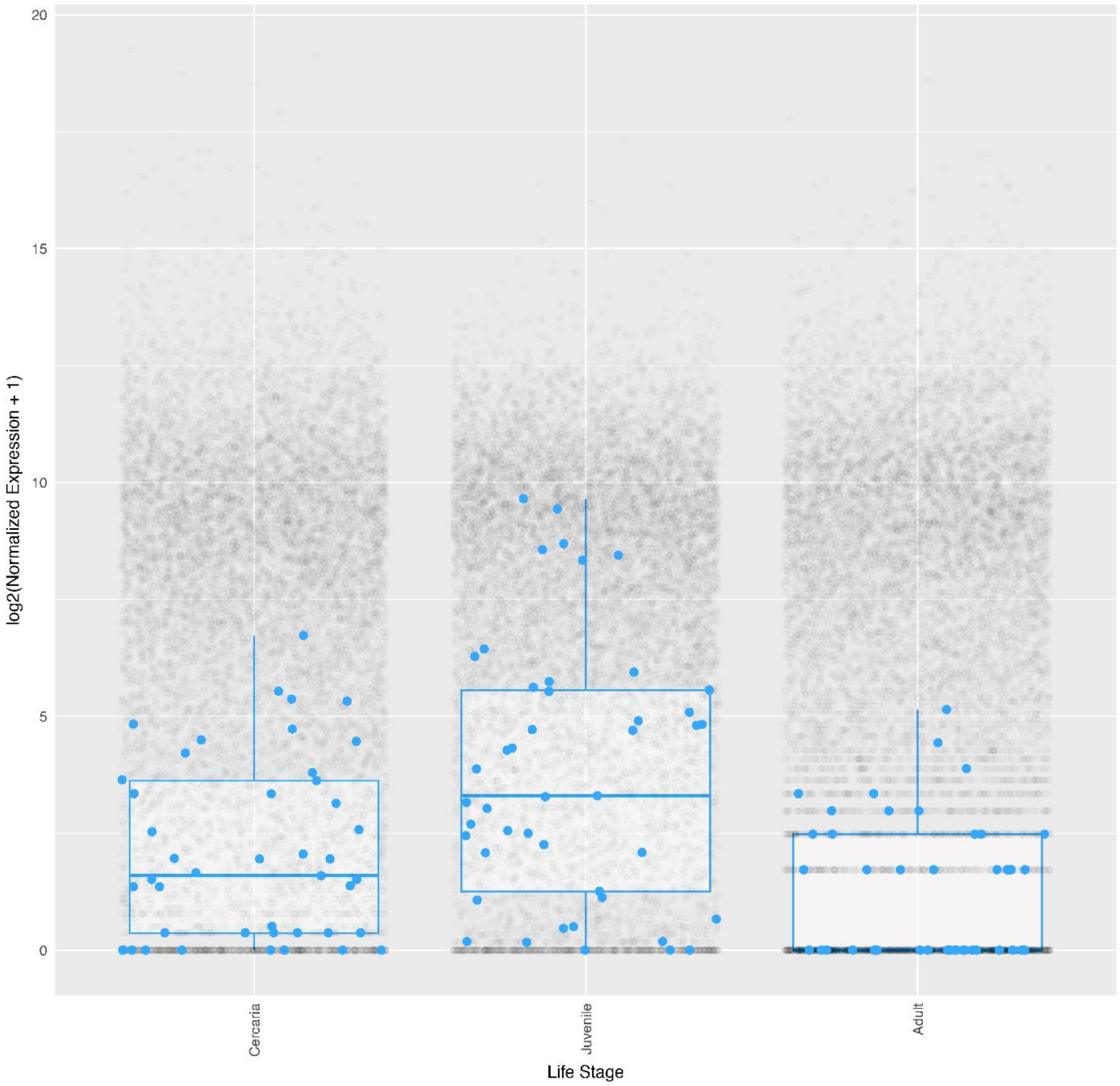

47 putative schistosome GPCRs were not captured in the Lu et al. 2016 dataset [32] (highlighted in blue). Analyzing the previously published stage-specific RNA-seq data [36] showed that the missing GPCRs were low abundantly transcribed in schistosome adults compared to other life stages, which explains their absence in this data set.

Figure S1: *F. hepatica* orthologs of GPCRs absent in in the underlying RNA-seq dataset are more abundantly transcribed in larvae and early juveniles.

B

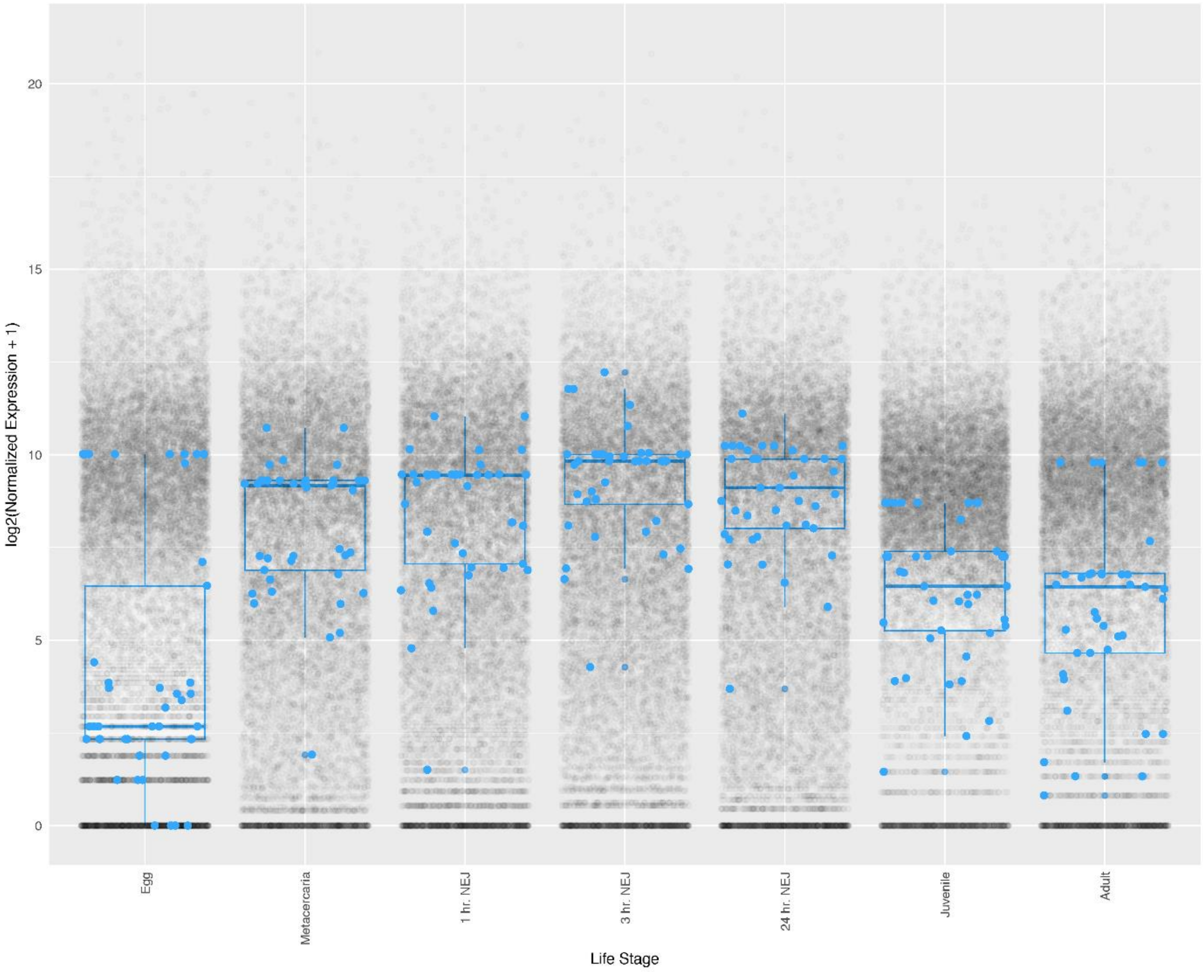

Orthologs of the 47 putative schistosome GPCRs that were not captured in the Lu et al. 2016 dataset [32] were analyzed for their transcript levels in different *F. hepatica* life stages (highlighted in blue) using previously published stage-specific RNAseq data [80]. While the *S. mansoni* orthologs showed low transcript levels in adults compared to other life stages, this pattern is less pronounced in liver flukes.
