# Supplementary material for: Tissue-specific transcriptome analyses provide new insights into GPCR signalling in adult Schistosoma mansoni
Source: PLoS Pathog. 2018 Jan 18;14(1):e1006718. doi: 10.1371/journal.ppat.1006718 (PMC5773224; doi:10.1371/journal.ppat.1006718)
Supplement: S2 Fig — GPCR, G protein–coupled receptor. (PDF) [file ppat.1006718.s003.pdf]

Figure S2: Transcript occurrence of GPCR orthologs in *F. hepatica* and *S. mansoni* adult stages.

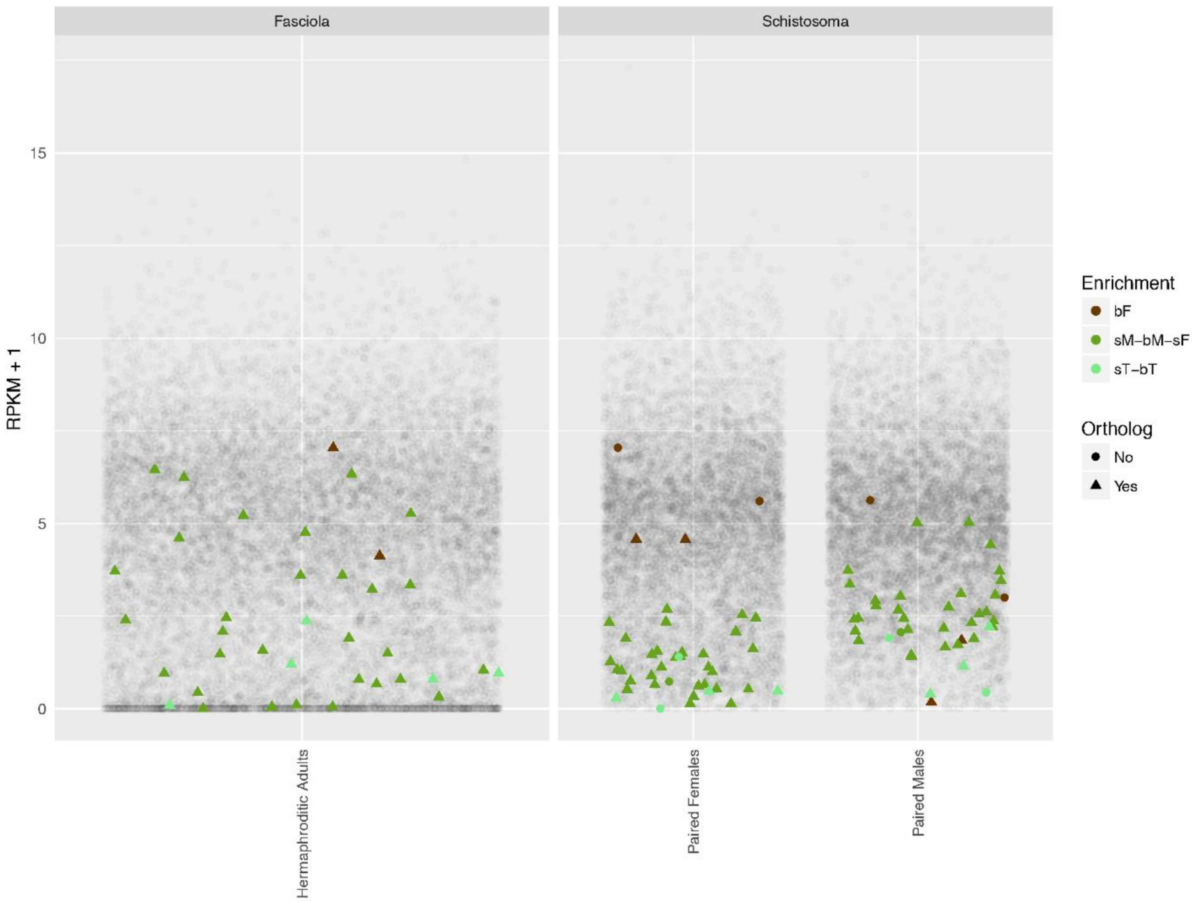

Transcript levels of GPCRs with a potential relevance in reproduction were shown for the mature stages of the dioecious trematode *S. mansoni* and the hermaphrodite trematode *F. hepatica* using recently published RNAseq data sets [32, 80]. Orthologs shared by both species are shown as triangles. Orthologs of the bF-group are among the most abundantly transcribed GPCRs in adult *F. hepatica and* female *S. mansoni,* while GPCRs of the sM-bM-sF group showed a wider range of expression in the hermaphrodite compared the dioecious stages. GPCRs of the sT-bT group exhibited generally low transcript levels. This is explained by the small fraction of testis tissue in adult trematodes. The occurrence of male gonad-associated transcripts in the female, and *vice versa*, indicate additional biological functions of these GPCRs.
